# Common issues raised during the quality assurance process of WHO guidelines: a cross-sectional study

**DOI:** 10.1186/s12961-018-0288-y

**Published:** 2018-02-07

**Authors:** Teegwendé V. Porgo, Mauricio Ferri, Susan L. Norris

**Affiliations:** 1 Population Health and Optimal Health Practices Research Unit, Department of Social and Preventative Medicine, Faculty of Medicine, Université Laval – Centre de Recherche du CHU de Québec, Hôpital de l’Enfant-Jésus, 1401 18e rue, H041, Quebec City, Quebec G1J 1Z4 Canada; 2 Guidelines Review Committee Secretariat, World Health Organization, Avenue Appia 20, 1211 Geneva 27, Switzerland

**Keywords:** WHO, Guidelines Review Committee, guideline development, evidence translation, quality assurance

## Abstract

**Background:**

In 2007, WHO established the Guidelines Review Committee (GRC) to ensure that WHO guidelines adhere to the highest international standards. The GRC reviews guideline proposals and final guidelines. The objectives of this study were to examine the rates of and reasons for conditional approval and non-approval of documents submitted for the first time to the GRC, and calculate the time intervals and numbers of submissions to achieve approval for documents conditionally approved or not approved at first submission.

**Methods:**

All initial submissions to the GRC between 2014 and 2017 were examined. Data were extracted from the GRC’s records of written comments and discussions.

**Results:**

Of a total of 85 proposals and 88 final guidelines, 32 (37.6%) proposals and 37 (42.0%) final guidelines were conditionally approved, and 15 (17.6%) proposals and 28 (31.8%) final guidelines were not. For both conditionally approved and not approved proposals, the most frequent reasons were suboptimal composition or inadequate description of the guideline contributor groups (in all proposals), followed by inadequate formulation of key questions (in 90.6% of conditionally approved proposals and all not approved proposals). For both conditionally approved and not approved final guidelines, the most frequent reasons were problems with recommendations (in all final guidelines), followed by inappropriate methods for evidence retrieval or an inadequate description thereof (in all conditionally approved final guidelines and 75.0% of not approved final guidelines). The median time to achieve approval was 2 months for proposals and 1–2 months for final guidelines. The median number of submissions was 2 for proposals and 2–2.5 for final guidelines.

**Conclusion:**

The GRC implements a rigorous quality assurance process and identifies problems with a significant percentage of initial submissions. WHO needs to continuously evaluate its guideline development processes to inform effective quality improvement measures and optimise the quality of its guidelines.

## Background

WHO guidelines are documents, whatever their title, that contain one or more recommendation(s) regarding health interventions or policies [1]. Recommendations are statements describing what policy-makers, healthcare providers, patients and other stakeholders should or could do to achieve the best health outcomes possible. WHO guidelines aim to enable end-users to make informed decisions regarding clinical or public health uncertainties. WHO guidelines address a broad range of topics, for example, environmental exposures, health systems, nutrition, patient safety, mental health, maternal and child health, infectious disease management, and public health emergency response [2].

In 2007, the WHO Director-General established the Guidelines Review Committee (GRC) [3] to ensure that WHO guidelines adhere to the highest international standards and are developed through a transparent and evidence-based decision-making process [1]. The GRC is composed of WHO staff and external members who serve 3-year terms. The committee functions as a peer-review body at two stages during the guideline development process, namely proposal and final guideline. Guideline proposals are assessed for scope, methods, appropriateness of group composition and feasibility. Final guidelines are assessed for adherence to the standards outlined in the *WHO Handbook for Guideline Development*, 2^nd^ edition [1], and for quality in the execution of the required development steps.

At monthly, closed meetings, the GRC makes its decisions via consensus, based only on information in the submitted documents. The assessment is one of approval, conditional approval or non-approval. Conditionally approved documents usually require revisions that can be addressed by the authors with oversight by the GRC Secretariat and the Chair, but without the need for another full GRC review. Documents that are not approved require significant revisions and the revised documents must be discussed at a subsequent GRC meeting. For both conditionally approved and not approved documents, authors must submit a revised document and provide point-by-point responses to the GRC’s comments.

The objectives of this study were to examine the rates of and reasons for conditional approval and non-approval of documents submitted for the first time to the GRC, and calculate the time intervals and numbers of submissions to achieve approval for documents conditionally approved or not approved at first submission.

## Methods

### Inclusion criteria

All proposals and final guidelines that were submitted for the first time for review at a GRC meeting between 1 January 2014 and 31 December 2017 were eligible for this study. All included documents were related to standard guidelines, and none was developed using abbreviated processes and methods in response to a public health emergency.

### Information sources

Data for this study were extracted from the GRC’s records of written comments from reviewers and from discussions during GRC meetings.

### Analyses

We examined trends in initial conditional approval and non-approval of proposals and final guidelines across the years using Cochran–Armitage trend tests for proportions, using the Statistical Analysis System (SAS Institute Inc., Cary, NC, USA, version 9.4). Differences were considered significant at *P* < 0.05.

Reasons for conditional approval and non-approval were categorised according to their primary focus, namely introduction and guideline scope; key questions underpinning the recommendations (in the Population, Intervention, Comparator, Outcome (PICO) format); guideline contributor group composition, roles and responsibilities; declarations of interest, management of conflict of interest and funding sources; methods for literature reviews; assessment of the certainty of the evidence; and formulation of recommendations.

The time interval and number of submissions to achieve approval were calculated for documents that had been approved as of January 15, 2018. The time interval to achieve approval for documents conditionally or not approved at first submission was calculated using the period between the GRC meeting date when a document was first reviewed and the date when the GRC approved the document. The decisions of the GRC are consistently provided 2 days after monthly meetings, and between-meeting approval of conditionally approved documents occurs within 2 to 3 days of submission. The number of submissions to achieve approval was calculated by counting the initial submission plus the number of subsequent submissions until approval was granted.

## Results

Between 2014 and 2017, 85 new proposals and 88 new final guidelines were submitted to the GRC (Table 1). Of these, 32 (37.6%) proposals and 37 (42.0%) final guidelines were conditionally approved, and 15 (17.6%) proposals and 28 (31.8%) final guidelines were not.

**Table 1:** Number and disposition of first-time submissions to the Guidelines Review Committee in 2014–2017

Year	Disposition, n (%)		
	Approved	Conditionally approved	Not approved
Guideline proposals (*n* = 85)			
2014	7 (38.9)	9 (50.0)	2 (11.1)
2015	6 (46.2)	5 (38.5)	2 (15.4)
2016	13 (52.0)	10 (40.0)	2 (8.0)
2017	12 (41.4)	8 (27.6)	9 (31.0)
Total	38 (44.7)	32 (37.6)	15 (17.6)
		*P* value = 0.17^a^	*P* value = 0.11^a^
Final guidelines (*n* = 88)			
2014	2 (10.5)	10 (52.6)	7 (36.8)
2015	4 (21.1)	8 (42.1)	7 (36.8)
2016	8 (27.6)	12 (41.4)	9 (31.0)
2017	9 (42.9)	7 (33.3)	5 (23.8)
Total	23 (26.1)	37 (42.0)	28 (31.8)
		*P* value = 0.28^a^	*P* value = 0.34^a^

^a^Cochran–Armitage trend tests

*n* number

### Rate of conditional approval and non-approval

The rate of conditional approval was between 27.6% (for proposals in 2017) and 52.6% (for final guidelines in 2014) and that for non-approval was between 8.0% (for proposals in 2016) and 36.8% (for final guidelines in 2014–2015). We did not observe a significant association between time and the rates of conditionally approved documents (*P* value for trend = 0.11–0.17) and not approved documents (*P* value for trend = 0.28–0.34).

### Reasons for initial conditional and non-approval

The reasons for initial conditional approval and non-approval are outlined in Table 2. For both conditionally approved and not approved proposals, the most frequent reasons were suboptimal composition or inadequate description of the guideline contributor groups (in all proposals), followed by inadequate formulation of key questions (in 90.6% of conditionally approved proposals and all not approved proposals). For both conditionally approved and not approved final guidelines, the most frequent reason was problems with recommendations (in all final guidelines), including unclear rationale for the strength of recommendations (in 75.7% of conditionally approved final guidelines and 92.9% of not approved final guidelines). The second most frequent problem noted in both conditionally approved and not approved final guidelines was inappropriate methods for evidence retrieval or an inadequate description thereof (in all conditionally approved final guidelines and 75.0% of not approved final guidelines).

**Table 2:** Reasons for initial conditional and non-approval of submissions to the Guidelines Review Committee in 2014–2017

Reasons	*n* (%)	
	Conditionally approved	Not approved
Guideline proposals^a^		
Problematic introduction and scope	25 (78.1)	14 (93.3)
Inadequate formulation of the key (‘PICO’) questions underpinning the recommendations	29 (90.6)	15 (100)
Suboptimal composition or inadequate description of the guideline contributor groups	32 (100)	15 (100)
WHO departments: inadequate representation or lack of clarity in staff members’ roles	19 (59.4)	10 (66.7)
GDG: inadequate diversity with respect to gender, WHO regions, low- and middle-income countries, technical expertise, and stakeholder perspectives or lack of clarity in members’ roles	29 (90.6)	15 (100)
Systematic review team, methodologist: inadequate description or lack of clarity in their roles	13 (40.6)	11 (73.3)
Concerns regarding reporting or assessment of DOI, management of COI or funding sources or inadequate description thereof	20 (62.5)	13 (86.7)
Inadequate methods for evidence retrieval or inadequate description thereof^b^	24 (75.0)	14 (93.3)
Inadequate description of the considerations for formulating recommendations^c^	29 (90.6)	14 (93.3)
Inadequate description of how values and preferences will be examined and inform the recommendations, including the perspectives that will be considered	7 (21.9)	7 (46.7)
Final guidelines^a^		
Suboptimal composition or inadequate description of the guideline contributor groups	21 (56.8)	18 (64.3)
WHO departments: inadequate representation or lack of clarity in staff members’ roles	5 (13.5)	4 (14.3)
GDG: inadequate diversity with respect to gender, WHO regions, low- and middle income countries, technical expertise, and stakeholder perspectives or lack of clarity in members’ roles	17 (46.0)	9 (32.1)
External review group: inadequate diversity with respect to gender, WHO regions, technical expertise, stakeholder and consumer representation or lack of clarity in members’ roles	11 (29.7)	11 (39.3)
Concerns regarding reporting or assessment of DOI, management of COI or funding sources or inadequate description thereof	20 (54.1)	19 (67.9)
Inadequate methods for evidence retrieval or inadequate description thereof^d^	37 (100)	21 (75.0)
Inadequate information on quality assessment of the evidence^e^	34 (91.9)	20 (71.4)
Problems with recommendations	37 (100)	28 (100)
Inadequate description of the expert group’s decision-making process^f^	17 (46.0)	16 (57.1)
Inadequate consideration of the key factors relevant to the decision-making process^g^	32 (86.5)	23 (82.1)
Unclear rationale for the strength of recommendation(s)	28 (75.7)	26 (92.9)
Suboptimal wording or content of the recommendation(s)	27 (73.0)	20 (71.4)
Inadequate considerations of the contextual factors associated with recommendation(s)	29 (29.7)	6 (21.4)

^a^Total: 32 conditionally approved and 15 not approved guideline proposals; 37 conditionally approved and 28 not approved final guidelines

^b^Draft search strategy and approach to quality assessment of individual studies and data synthesis

^c^Methods for GRADE certainty of evidence and strength of recommendation, use of existing guidelines

^d^Literature review(s) not available to the Guidelines Review Committee, methods unclear, suboptimal criteria for study inclusion/exclusion, inadequate quality assessment of individual studies or data synthesis and interpretation, outdated bibliographic database search

^e^Incomplete description of how the body of evidence was assessed, GRADE evidence profiles not provided or problematic

^f^Voting, definition of consensus and majority, management of disagreements

^g^Acceptability, resource use, costs and cost-effectiveness, values and preferences, benefits and harms

Note that each proposal or guideline can have multiple reasons for conditional approval or non-approval

*n* number, *PICO* Population Intervention Comparator Outcome, *GDG* guideline development group, *DOI* declarations of interest, *COI* conflicts of interest, *GRADE* Grading of Recommendations Assessment, Development and Evaluation

### Time interval and number of submissions to achieve approval

Among documents that were conditionally approved or not approved at initial submission in 2014–2017, 34 (72.3%) proposals and 58 (89.2%) final guidelines had been approved by January 15, 2018. The median time to achieve approval was 2 months for proposals and 1–2 months for final guidelines (Table 3). The median number of submissions before approval was 2 for proposals and 2–2.5 for final guidelines. Among the 19 documents (13 proposals and 6 final guidelines) that had not yet been approved, 14 had been submitted once, 4 had been submitted twice and 1 three times. One document was initially submitted in 2014, 2 in 2015, 4 in 2016 and 12 in 2017.

**Table 3:** Time intervals and number of submissions needed to achieve approval for documents conditionally or not approved at first submission in 2014–2017

	Median (Minimum–Maximum)	
	Conditionally approved	Not approved
Guideline proposals^a^		
Time (months)	2 (1–7)	2 (1–5)
Number of submissions	2 (2–2)	2 (2–3)
Final guidelines^a^		
Time (months)	1 (1–6)	2 (1–13)
Number of submissions	2 (2–2)	2.5 (2–4)

*n*, number

^a^As of January 15, 2018, the Guidelines Review Committee subsequently approved 34 (72.3%) guideline proposals (27 (84.4%) conditionally approved and 7 (46.7%) not approved at the initial submission) and 58 (89.2%) final guidelines (34 (94.4%) conditionally approved and 24 (85.7%) not approved at the initial submission)

## Discussion

The GRC and its Secretariat provide WHO staff with technical advice and training for guideline development. Nonetheless, a significant proportion of proposals and final guidelines submitted to the GRC between 2014 and 2017 did not meet WHO’s standards and were either conditionally approved or not approved at first submission, leading to delayed approval and therefore delayed publication. Nearly all proposals had issues with the composition of the guideline contributor groups and the key (‘PICO’) questions. All final guidelines had concerns regarding the recommendations, which included an unclear rationale for the strength of recommendation, and more than three-quarters were submitted with issues regarding the reporting of methods for evidence retrieval. The majority of proposals and final guidelines also had issues with declarations of interest, management of conflicts of interest or funding sources. Half of the documents that were initially conditionally approved or not approved were subsequently approved after a second submission, and within 1 or 2 months. However, some documents required additional revisions over lengthy periods before approval was granted.

Planning proposals are generally short, concise documents that must comply with a reporting checklist provided by the GRC Secretariat. The Secretariat also provides a detailed, structured template for proposals since 2014. The GRC expects the proposal to convey that the guideline developers have clear, achievable objectives and know how to approach the guideline development steps, even if all the details are not provided. The high rate of concerns with the scope and key (‘PICO’) questions may be explained by the fact that some proposals are submitted at an early stage when the WHO steering group and the guideline development group have not finalised these decisions. Getting the scope and key questions right is critical as they represent the health issues that guidelines aim to address and form the basis of the evidence searches which underpin the recommendations. Consequently, key questions must be finalised early in the guideline development process and the GRC Secretariat is working with WHO staff to help ensure that key questions are clear, answerable and acceptable to a wide range of stakeholders.

Furthermore, other problems, such as suboptimal composition of the guideline contributor groups and concerns regarding funding sources, declarations of interest or management of conflicts of interest, also need to be addressed at the planning stage since they are irremediable at the final guideline stage. To better address this issue, the GRC instituted a policy in 2017 that requires GRC Secretariat review of proposed guideline development group members, their declarations of interest and the management plans for any conflicts of interest before the meeting at which recommendations are formulated.

With regard to final guidelines, the GRC noted particular problems with the rationale statement for recommendations. Rationale statements should be clear, concise and cogent statements that articulate the basis for the recommendation, encompassing the balance of benefits and harms as well as considerations of equity, human rights, acceptability, resource use, and feasibility, among others, as relevant. These statements are essential for a high-quality and transparent guideline. Potential solutions include more engagement of the guideline methodologist, whose main role is to assist the guideline development group in formulating recommendations based on evidence. The methodologist may have additional roles, including helping to develop key questions, reviewing the systematic review team’s assessments of the certainty of evidence, helping to draft the methods section of the final guideline, and reviewing the draft final guideline [1]. Additional solutions include staff training on using an evidence-to-decision framework such as GRADE-DECIDE [4], which delineates a comprehensive list of the key elements (in addition to benefits and harms of the interventions) that should underpin recommendations. This will help guide searches for relevant evidence at the beginning of the final guideline development process as well as the guideline development group’s discussions and the subsequent rationale statement for each recommendation. Problems noted with documentation and methods of the evidence reviews are being addressed with additional staff training, more attention to the terms of reference for commissioned systematic reviews and increased contact between WHO staff and contractors. In addition, WHO staff are linked to WHO information scientists who have extensive experience with systematic reviews of public health interventions, including the grey literature.

WHO’s quality assurance process is rigorous and self-evaluation efforts such as this study feed back into quality improvement efforts. The high rate of conditional approval and non-approval of both planning proposals and final guidelines is concerning, but presents clear opportunities for quality improvement such as targeted training of guideline developers, dissemination of best practice examples, question-and-answer sessions, and more individual and group consultations with the GRC Secretariat, among other possible strategies.

An important strength of this study is that we had access to all documents submitted to the GRC during the time period examined, as well as to all comments provided by the GRC and its Secretariat during the review process. Nonetheless, several limitations should be noted. First, there are many factors that influence the number and nature of the comments on documents and the decisions made by the GRC. Like any peer-review process, comments vary across reviewers and over time within each reviewer, and GRC members generally serve 3-year terms. Second, the experience and training of WHO technical units, staff and the GRC Secretariat increased over time and an updated, more detailed *WHO Handbook for Guideline Development*, 2^nd^ edition, was published in December 2014 [1] and widely disseminated to WHO staff developing guidelines. Additional considerations were added to the 2014 handbook such as attention to human rights, equity and social determinants of health, including gender. Third, the categories of reasons for conditional approval and non-approval vary in scope and level of detail; thus, it is not appropriate to compare the number of problematic documents across categories. The categorisation of reasons for non-approval is also a rather subjective process. Fourth, because of the small numbers of documents, the study has low statistical power to show trends in rates of approval over time. Finally, this study was conceived, implemented and written by three individuals with an affiliation to WHO, representing a potential source of bias particularly in the interpretation of the results. We tried to minimise the risk of bias by having the external author (TVP) perform all data extraction and analyses (with subsequent checking by the other authors).

### Implications for other organisations that develop guidelines

The processes, methods and standards that are implemented by the GRC are exemplary and relevant to every organisation that develops guidelines. WHO has implemented and executed a transparent, efficient and sustainable quality assurance process for all of its guidelines consisting of structured peer review, actionable constructive feedback, and technical and process support and training. Guideline development groups might consider whether the model of WHO’s GRC might be adapted to their settings and needs. The reasons for non-approval of guideline proposals and final guidelines that we identified are likely relevant to documents developed by other organisations since WHO’s procedures and methods are consistent with those of the international guideline community, and WHO uses existing high-quality systematic reviews and commissions new reviews from external teams. Thus, the weaknesses identified at first review of WHO documents are also likely relevant to many other organisations.

## Conclusions

The WHO GRC implements a rigorous quality assurance process and identifies problems with a significant percentage of documents submitted for the first time. The reasons for conditional approval and non-approval of proposals were related to fundamental steps in the guideline development process that must be remedied early. For final guidelines, the reasons pertained most commonly to the recommendations and the lack of a clear linkage to the evidence and other considerations. WHO needs to continuously evaluate its guideline development processes and outputs, and use that information to inform effective quality improvement measures. This will help to ensure that WHO’s normative guidance to United Nations Member States will optimally impact global public health.

## Abbreviations

DECIDE: Developing and Evaluating Communication strategies to support Informed Decisions and practice based on Evidence
GRADE: Grading of Recommendations, Assessment, Development and Evaluation
GRC: Guidelines Review Committee
PICO: Population, Intervention, Comparator, Outcome
